# Morphological and Transcriptomic Analyses Reveal the Toxicological Mechanism and Risk of Nitrate Exposure in *Bufo gargarizans* Embryos

**DOI:** 10.3390/ani14060961

**Published:** 2024-03-20

**Authors:** Lei Xie, Ziyi Niu, Shimin Xiao, Hongyuan Wang, Yongpu Zhang

**Affiliations:** 1 Life and Environmental Science College, Wenzhou University, Wenzhou 325003, China; 2 Zhejiang Provincial Key Laboratory for Subtropical Water Environment and Marine Biological Resources Protection, Wenzhou University, Wenzhou 325003, China; 3 College of Life Science, Shaanxi Normal University, Xi’an 710119, China

**Keywords:** nitrate, anuran, embryo, morphological parameters, transcriptomics

## Abstract

**Simple Summary:**

The ingestion of excessive nitrate can affect the thyroid gland and cause thyroid dysfunction in humans. In the present study, amphibian embryos were exposed to nitrate, thyroxine and methimazole (a thyroid peroxidase inhibitor) during embryonic development to further explore the effects of nitrate on the thyroid. The results showed that nitrate, thyroxine and methimazole inhibited embryo growth and development. Additionally, methimazole and high concentrations of nitrate downregulated the genes related to thyroid morphogenesis and cholesterol metabolism, while upregulating the genes related to inflammation and apoptosis. These suggested that nitrate not only damaged the thyroid gland, but also affected the formation of the thyroid, thus affecting embryonic development.

**Abstract:**

In recent years, nitrate (NO_3_-N) pollution in water bodies has been increasing due to the excessive use of nitrogen-based fertilizers. Exposure to NO_3_-N during the development of amphibian embryos may have lasting effects on the growth and development of individuals and even threaten their survival, but the toxicity mechanism of NO_3_-N in amphibian embryos prior to thyroid morphogenesis remains unclear. In the present study, *Bufo gargarizans* was selected as the model organism to investigate the toxic effects of 10 mg/L and 100 mg/L NO_3_-N exposure (N10 and N100) on amphibian embryos using methimazole (MMI) and exogenous thyroxine (T4) as the reference groups. We found that T4, MMI, N10 and N100 inhibited *B. gargarizans* embryo growth and development, with MMI and N100 showing the earliest and strongest effects. Transcriptome analysis revealed that MMI and NO_3_-N (especially N100) significantly downregulated genes related to thyroid morphogenesis and cholesterol metabolism, while upregulating genes related to inflammation and apoptosis. Together, these results contribute to a deeper understanding of the complex mechanisms by which NO_3_-N disrupts *B. gargarizans* embryonic development, reveal the potential risks of NO_3_-N pollution to other aquatic organisms, and provide insights into the conservation of a broader ecosystem.

## 1. Introduction

Nitrate pollution in water is a widespread global problem and has received increasing attention in recent decades due to its adverse effects on aquatic organisms, human health, and ecosystems [1,2]. The primary cause of nitrate pollution is human agricultural activities, excessive application of nitrogen-based fertilizers and manure, which can transport nitrates to rivers, lakes and groundwater through runoff, resulting in elevated nitrate levels in water bodies [3]. In addition, an inadequate treatment of sewage and wastewater from various industries, such as tanning, paper making and mining, can also significantly contribute to nitrate pollution [4]. The permissible limit of nitrate–nitrogen (NO_3_-N) in drinking water is 10 mg/L according to the U.S. Environmental Protection Agency (USEPA), and 11.3 mg/L according to the World Health Organization (WHO) guidelines. However, the concentration of NO_3_-N in many waters already far exceeds this standard. For example, the NO_3_-N concentrations range from 55 to 231mg/L in groundwater in the Grombalia Basin (Tunisia) and can reach 153.29 mg/L in the Naoli River in the Sanjiang Plain in China [5,6].

The detrimental impacts of excessive NO_3_-N in water systems on aquatic organisms have been reported in previous research [7]. For instance, exposure to high NO_3_-N levels has been linked to stunted growth, compromised health status and disorders in immune and lipid metabolism in juvenile turbot (*Scophthalmus maximus*) [2,8]. In addition, decreased sperm counts and increased testicular weight in mosquito fish (*Gambusia holbrooki*) were associated with increased NO_3_-N concentrations [9]. Chronic exposure to NO_3_-N disrupts the synthesis and metabolism of steroid hormones in fathead minnows (*Pimephales promelas*), ultimately affecting their reproduction and survival [10]. Moreover, increased NO_3_-N concentrations significantly diminish the feed intake and growth rates of African catfish (*Clarias gariepinus*) [11].

Amphibian breeding habitats often overlap with agricultural lands, making them particularly susceptible to exposure to NO_3_-N, a key constituent of fertilizers used in farming [12,13]. Unlike adult amphibians, which can move between habitats, the mobility of embryonic amphibians is restricted, hindering their ability to evade or escape NO_3_-N-contaminated environments [14]. The embryonic period is a pivotal phase in the development of amphibian organs and systems, and exposures during embryonic development potentially exert enduring impacts on an individual’s growth, development and overall health [15,16]. Prior research into NO_3_-N exposure in amphibians has predominantly centered on its disruption of the thyroid axis and resultant thyroid dysfunction, including the depletion of thyroid follicular colloids and reduced thyroid hormone (TH) levels [17,18,19]. It is important to note, however, that the morphogenesis of the amphibian thyroid gland do not occur until around Nieuwkoop and Faber (NF) stage 43 (approximated by Gosner (Gs) stage 23) [20,21,22], and only maternal thyroid hormones function before then [23]. Consequently, we raise two scientific questions here: (1) Does NO_3_-N influence the expression levels of thyroid-related genes during the embryonic stage when the thyroid gland has not undergone morphogenesis? (2) If NO_3_-N does not impact the expression of thyroid-related genes, what mechanisms underlie the adverse effects of NO_3_-N on the embryo?

*Bufo gargarizans*, a species of amphibian belonging to the family Bufonidae, is native to East Asia, including China, Korea and Japan [24]. They inhabit areas prone to NO_3_-N accumulation, including woodlands, wetlands, ponds and agricultural fields, and generally undergo “explosive breeding” during the spring [25,26]. Due to their high fertility and highly permeable skin and gills, *B. gargarizans* is considered a good model for understanding the toxicity of aquatic environmental pollutants [27]. Methimazole (MMI) is a well-characterized inhibitor of thyroid peroxidase, inhibiting thyroid hormone (TH) synthesis [28]. This study used the MMI and TH enhancer exogenous thyroxine (T4) as the reference groups [29], and established low-concentration (10 mg/L) and high-concentration (100 mg/L) NO_3_-N exposure groups for experiments. Through morphological measurements and RNA-Seq combined with bioinformatics analysis, we investigated the potential mechanism of NO_3_-N toxicity to embryos, aiming to contribute to assessing their environmental and ecological risks.

## 2. Materials and Methods

### 2.1. Experimental Solution

Methimazole (MMI, CAS: 60-56-0, purity 98%), exogenous L-thyroxine (T4, CAS: 51-48-9, purity 98%) and sodium hydroxide (NaOH, CAS No. 1310-73-2, purity 95%) were purchased from Aladdin (Shanghai, China). Sodium nitrate (NaNO_3_, purity 99%, CAS: 7631-99-4) was purchased from Sigma-Aldrich Corporation (Sigma, St. Louis, MO, USA). A stock solution of 80 mg/L MMI was prepared by dissolving 0.08 g MMI in 1 L distilled water, while a stock solution of 10 μg/L T4 was prepared in 0.1 N sodium hydroxide solutions buffered with 0.1 N muriatic acid solutions as solvents. The final concentrations of T4 (2 μg/L) and MMI (8 mg/L) were obtained via diluting stock solutions with dechlorinated water. A stock solution of 1000 mg/L NO_3_-N was prepared by dissolving 6.07 g NaNO_3_ in 1 L of distilled water, and working solutions of 10 mg/L and 100 mg/L NO_3_-N were obtained via diluting stock solutions with dechlorinated water. Selected concentrations of NO_3_-N for the experiment were within environmental ranges.

### 2.2. Animal Husbandry and Exposure Experiment

A pair of mating *B. gargarizans* were captured from Qinling Mountains of Shaanxi Province in China and raised in glass tanks with dechlorinated water (pH = 6.8 ± 0.3) until they spawned. Fertilized eggs were kept in fresh dechlorinated water until the Gs3 stage, after which they were randomly assigned to a glass tank (50 cm × 20 cm × 20 cm) with 1.5 L treatment solutions with T4, MMI, 0, 10 and 100 mg/L NO_3_-N. Three replicates were set up for each treatment group with 100 embryos per replicate. The experiment was conducted in tanks with a 12 h light/12 h dark photoperiod and a temperature of 21 ± 2 °C, and embryos were not fed. The test solution was replaced completely every 2 days to maintain exposure concentrations and the exposure was sustained for 11 days. Animal husbandry and experiments were approved by the Animal Ethical and Welfare Committee of Wenzhou University and were consistent with the guidelines of China Wildlife Conservation Association.

### 2.3. Embryonic Developmental Stage Determination and Morphologic Measurements

Embryo samples were collected for morphologic measurements and developmental staging on the 7th and 9th days after exposure (*n* = 5/replicate, *n* = 15/treatment, respectively). The embryos were fixed with 4% paraformaldehyde for 24 h and preserved in 70% ethanol. Body weight and body length of embryos were measured using an analytical balance with 0.1 mg readability and Tesa-Cal Dura-Cal Digital electronic calipers with 0.01 mm precision. Embryos were observed and photographed using a Zeiss Discovery V12 stereoscope equipped with a cannon 7D digital camera, and the embryonic development stage was determined according to the staging criteria proposed by Gosner (1960) [21].

### 2.4. Transcriptomic Analysis

On the eleventh day after exposure, the *B. gargarizans* embryos from five treatment groups (*n* = 30/replicate, *n* = 90/treatment) were frozen in liquid nitrogen and stored at −80 °C to generate the transcriptome. Total RNA was extracted from tissue using TRIzol Reagent (Invitrogen, Carlsbad, MA, USA) and treated with DNase I (TaKara, Shiga, Japan). The RNA quality and quantity were assessed using a 2100 Bioanalyzer (Agilent, Santa Clara, CA, USA) and ND-2000 (NanoDrop Technologies, Wilmington, DE, USA). Illumina HiSeq Sequencing libraries were prepared from 1 μg of total RNA using the TruSeqTM RNA sample preparation Kit (Illumina, San Diego, CA, USA). Libraries were quantified with a TBS380 and sequenced on an Illumina NovaSeq 6000 sequencer (150 bp×2, Shanghai BIOZERON Co., Ltd., Shanghai, China). Quality control was performed using Trimmomatic (version 0.36).

Clean data from all samples were subjected to RNA de novo assembly with Trinity. Assembled transcripts were BLASTX-searched against the NR, String, and Kyoto Encyclopedia of Genes and Genomes (KEGG) databases (E-value < 1.0 × 10^−5^). Gene Ontology (GO) annotations were obtained via BLAST2GO, and metabolic pathways were analyzed using KEGG. Transcript expression levels were quantified using RSEM, and differential expression genes (DEGs) were identified with EdgeR. Functional enrichment analyses (GO and KEGG) identified significantly enriched DEGs (Bonferroni-corrected *p*-value ≤ 0.05) and were conducted using Goatools and KOBAS.

### 2.5. Statistical Analysis

By using SPSS 26 software, significant differences between treatments were calculated based on a one-way analysis of variance (ANOVA) and least significant difference (LSD) post hoc test. The homogeneity of variance was tested using Levene’s test. Differences were considered significant when *p* < 0.05, and all data are presented as the mean ± SD. Graphs were drawn by Origin 2021 software.

## 3. Results

### 3.1. Embryonic Growth and Development

**Figure 1:**
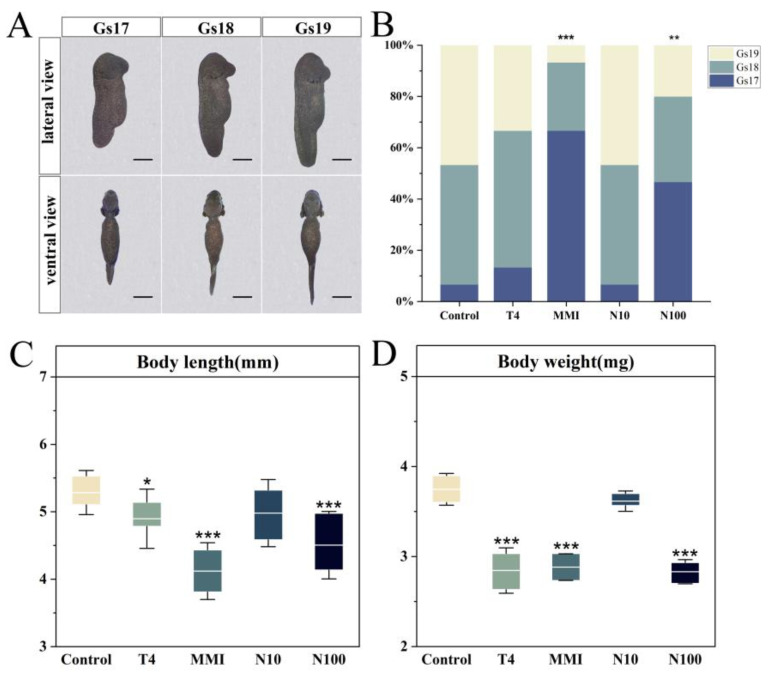
Effects of different exposure treatments (T4, MMI, N10, N100) on the growth and development of the *B. gargarizans* embryos on day 7 after exposure (*n* = 15). (**A**) Lateral and ventral views of embryos at Gs17-19. (**B**) Stages of development on day 7 and values are expressed as a percentage (%). (**C**) Body length. (**D**) Body weight. Bars represent the mean ± SD. Significant differences between the treatment and control groups are indicated by an asterisk, * *p* < 0.01, ** *p* < 0.01, *** *p* < 0.001.

We assessed the effects of nitrate, methimazole (MMI) and exogenous L-thyroxine (T4) on embryonic growth and development by measuring the developmental stage, body length and body weight of embryos. On the 7th day of exposure, the embryos were distributed between Gs17 and Gs19 (Figure 1A). Compared to the control group, the T4 and N10 had no significant effect on developmental stages (ANOVA, *p* > 0.05), but MMI and N100 significantly inhibited the developmental stages (ANOVA, *p* < 0.01) (Figure 1B). Of the embryos in the control group, 46.67% reached the Gs19 stage on day 7, compared to 6.67% in the MMI group and 20% in the N100 group. In addition, the body length and body weight of embryos were significantly reduced in the T4, MMI and N100 groups compared to the control group (ANOVA, *p* < 0.05) (Figure 1C,D).

**Figure 2:**
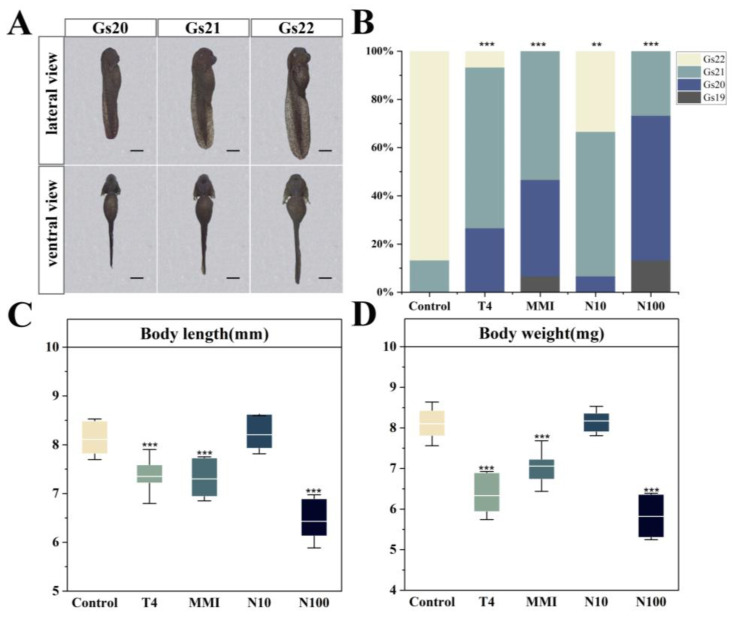
Effects of different exposure treatments (T4, MMI, N10, N100) on growth and development in the *B. gargarizans* embryos on day 9 after exposure (*n* = 15). (**A**) Lateral and ventral views of embryos at Gs20-22. (**B**) Stages of development on day 9 and values are expressed as a percentage (%). (**C**) Body length. (**D**) Body weight. Bars represent the mean ± SD. Significant differences between the treatment and control groups are indicated by an asterisk, ** *p* < 0.01, *** *p* < 0.001.

On the 9th day of exposure, the embryos spanned four developmental stages: Gs19, Gs20, Gs21 and Gs22 (Figure 2A). Compared to the control group, all treatment groups showed a significant inhibition of the developmental stages (ANOVA, *p* < 0.01) (Figure 2B). Most embryos in the control group (86.67%) reached Gs22 on day 9, compared to 6.67% and 33.33% in T4 and N10 groups, respectively, and no embryos in the MMI and N100 groups reached Gs22 on day 9. Moreover, both the body length and body weight of embryos were significantly reduced in the T4, MMI and N100 groups in comparison to the control group (ANOVA, *p* < 0.001) (Figure 2C,D).

### 3.2. Identification of Differentially Expressed Genes (DEGs)

**Figure 3:**
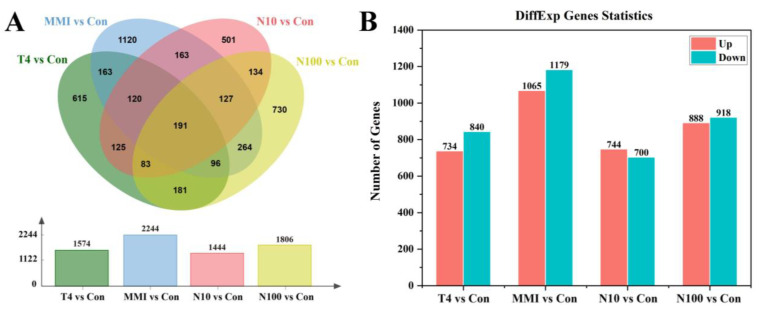
Differential expression of genes (DEGs) in embryos of *B. gargarizans* exposed to T4, MMI, N10, and N100. (**A**) Venn diagrams of DEGs for four comparisons: T4 vs. Con, MMI vs. Con, N10 vs. Con, N100 vs. Con. (**B**) The bar chart shows the number of upregulated and downregulated DEGs in the T4 vs. Con, MMI vs. Con, N10 vs. Con, N100 vs. Con groups.

To test the gene expression response of *B. gargarizans* embryos exposed to T4, MMI, N10 and N100, we filtered DEGs among control and treatment groups (Figure 3). Of the DEGS, 1574 were found between the T4 and Con groups, including 734 upregulated genes and 840 downregulated genes; 2244 DEGs were identified between the MMI and Con groups, including 1065 upregulated genes and 1179 downregulated genes; the N10 and Con groups showed 1444 DEGs, including 744 upregulated genes and 700 downregulated genes; and between the N100 and Con groups, a total of 1806 DEGs were found, of which, 888 were upregulated and 918 were downregulated (Figure 3A,B). Through the Venn diagrams to investigate the shared DEGs among the control and four treatment groups, we found that 615 DEGs were only in the T4 vs. Con group, 1120 DEGs were only in the MMI vs. Con group, 501 DEGs were only in the N10 vs. Con group, 730 DEGs were only in the N100 vs. Con group and 191 DEGs were shared by all the four treatment groups (Figure 3A). Notably, MMI vs. Con group and the N100 vs. Con group shared a total of 678 DEGs, which was the largest among the four groups.

### 3.3. GO and KEGG Enrichment

**Figure 4:**
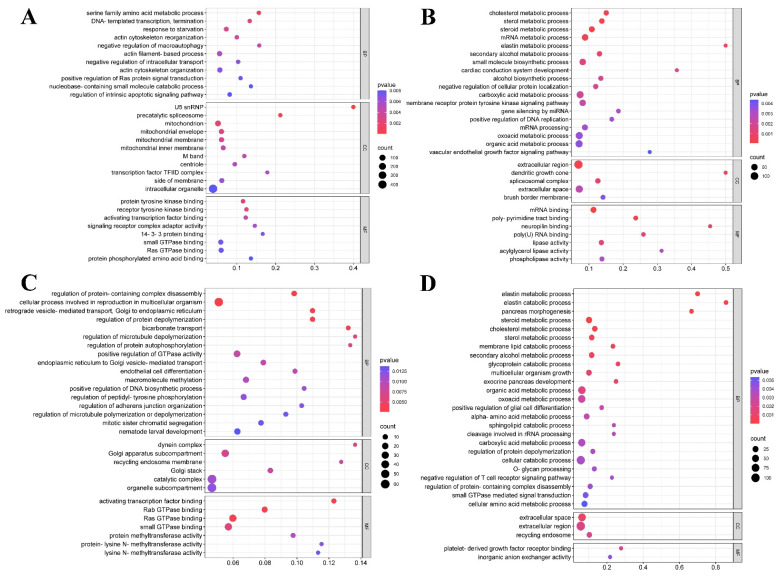
Bubble charts showing the the top 30 significant enrichment pathways of Gene Ontology (GO) classification for DEGs in (**A**) T4 vs. Con, (**B**) MMI vs. Con, and (**C**) N10 vs. Con, (**D**) N100 vs. Con groups of *B. gargarizans* embryos. The vertical axis of the bubble chats is the functional classification, and the horizontal axis is the rich factor, which is the ratio of DEG numbers annotated in a pathway to all those annotated in that pathway. The color of the bubble represents the enriched *p*-value and the size of the bubble represents the number of DEGs in the functional classification. BP, biological process; CC, cellular component; MF, molecular functions.

To further investigate the biological functions of these DEGs, GO category enrichment analysis was performed. The GO pathways are divided into three functional categories: BP (biological process), CC (cellular component) and MF (molecular functions). The top 30 GO pathways with the most significant enrichment in four pair-wise comparison groups were shown in Figure 4. In the T4 vs. Con group, DEGs were annotated into 11 pathways in the BP domain, such as serine family amino acid metabolic process and regulation of intrinsic apoptotic signaling pathway etc.; 11 pathways in the CC domain, including mitochondrial envelope and mitochondrial membrane, etc.; and 8 pathways in the MF domain, including small GTPase binding and protein tyrosine kinase binding, etc. (Figure 4A). In the MMI vs. Con group, DEGs were enriched in 18 pathways in the BP domain, such as cholesterol metabolic process, secondary alcohol metabolic process and organic acid metabolic process; 5 pathways in the CC domain, including extracellular region and extracellular space, etc.; 7 pathways in the MF domain, including mRNA binding and lipase activity, etc. (Figure 4B). In the N10 vs. Con group, DEGs were involved in 17 pathways in the BP domain, such as the regulation of microtubule depolymerization and protein autophosphorylation; 6 pathways in CC domain, including Golgi stack and recycling endosome membrane, etc.; 7 pathways in the MF domain, including protein methyltransferase activity and Ras GTPase binding, etc. (Figure 4C). In the N100 vs. Con group, DEGs were mainly enriched in BP domain (25 pathways), including elastin metabolic process, sterol metabolic process and small GTPase mediated signal transduction etc. DEGs were also enriched in extracellular space, extracellular region and recycling endosome in the CC domain, and platelet-derived growth factor receptor binding and inorganic anion exchanger activity in the MF domain (Figure 4D).

**Figure 5:**
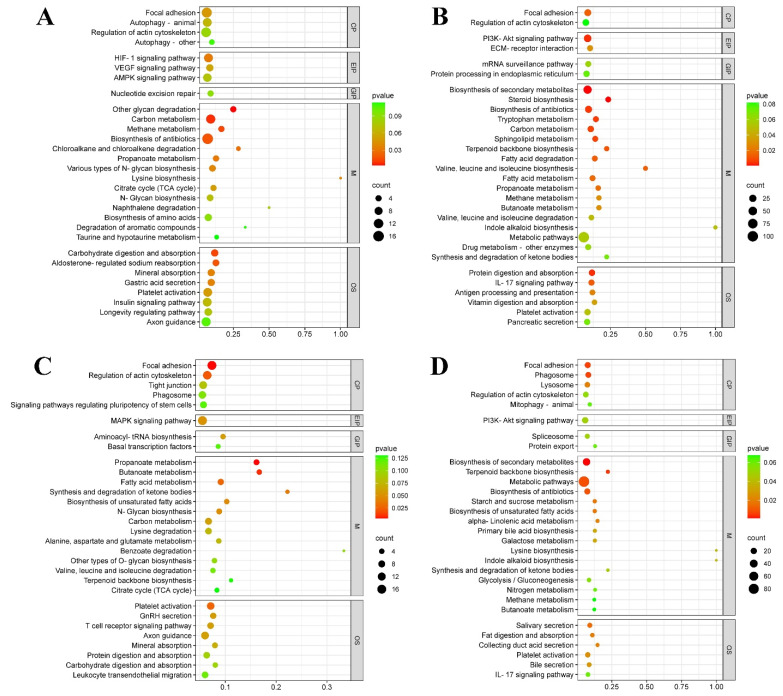
Bubble charts showing the top 30 significant enrichment pathways of the Kyoto Encyclopedia of Genes and Genomes (KEGG) for DEGs in (**A**) T4 vs. Con, (**B**) MMI vs. Con, and (**C**) N10 vs. Con, (**D**) N100 vs. Con groups of *B. gargarizans* embryos. The vertical axis of the bubble chats is the functional classification, and the horizontal axis is the rich factor, which is the ratio of DEG numbers annotated in a pathway to all those annotated in that pathway. The color of the bubble represents the enriched p-value and the size of the bubble represents the number of DEGs in the functional classification. M, metabolism; GIP, genetic information processing; EIP, environmental information processing; CP, cellular processes; OS, organizational systems.

To gain a better understanding of the biochemical metabolic pathways of these DEGs, KEGG analysis was conducted (Figure 5). The top 30 significant enrichment KEGG pathways were divided into five categories: metabolism (M), genetic information processing (GIP), environmental information processing (EIP), cellular processes (CP), and organizational systems (OS). In the T4 vs. Con group, KEGG enrichment results showed that 4, 3, 1, 14 and 8 terms were involved in CP, EIP, GIP, M, and OS, respectively (Figure 5A), including autophagy (CP), VEGF signaling pathway (EIP), nucleotide excision repair (GIP), carbon metabolism (M), and carbohydrate digestion and absorption (OS) etc. The DEGs in the MMI vs. Con group were enriched into focal adhesion (CP), PI3K-Akt signaling pathway (EIP), mRNA surveillance pathway (GIP), steroid biosynthesis (M) and the IL-17 signaling pathway (OS), etc. (Figure 5B). The DEGs in the N10 vs. Con group were enriched in many pathways, such as regulation of actin cytoskeleton (CP), MAPK signaling pathway (EIP), aminoacyl- tRNA biosynthesis (GIP), fatty acid metabolism (M) and T-cell receptor signaling pathway (OS) (Figure 5C). The N100 vs. Con group showed significant enrichment in pathways related to lysosome (CP), PI3K-Akt signaling pathway (EIP), protein export (GIP), nitrogen metabolism (M), fat digestion and absorption (OS), etc. (Figure 5D).

**Figure 6:**
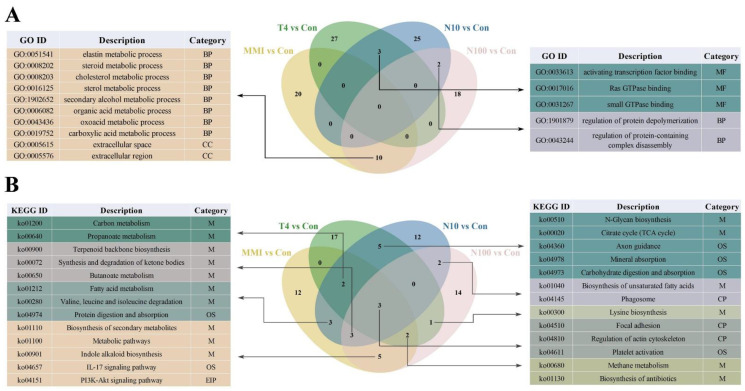
Venn diagrams and annotation of the common pathways in the top 30 significant enrichment pathways for four pair-wise comparison groups (T4 vs. Con, MMI vs. Con, N10 vs. Con, and N100 vs. Con). (**A**) GO. Molecular function (MF), biological process (BP), and cellular component (CC). (**B**) KEGG. Metabolism (M), genetic information processing (GIP), environmental information processing (EIP), cellular processes (CP), and organizational systems (OS).

The Venn diagram further showed the intersection of the top 30 enriched GO and KEGG functional pathways in four pair-wise comparison groups (Figure 6). In the GO analysis (Figure 6A), the MMI vs. Con group and N100 vs. Con group simultaneously significantly enriched 10 pathways. It is worth noting that there were three pathways involved in sterol metabolism, including steroid metabolic process, cholesterol metabolic process and sterol metabolic process. The T4 vs. Con group and N10 vs. Con group shared three enrichment pathways related to GTPase, including Ras GTPase binding, small GTPase binding and activating transcription factor binding. The N10 vs. Con group and N100 vs. Con group shared two enrichment pathways related to protein, including the regulation of protein depolymerization and protein-containing complex disassembly. In the KEGG analysis (Figure 6B), the highest number of overlapping pathways (13 pathways) was found between the MMI vs. Con and N100 vs. Con groups among the four pair-wise comparison groups. There were eight pathways associated with metabolism, such as the synthesis and degradation of ketone bodies, butanoate metabolism, methane metabolism; two pathways were immune system related, including IL-17 signaling pathway and platelet activation; one pathway related to signal transduction was the PI3K-Akt signaling pathway; two pathways were involved in cellular processes, which were the regulation of actin cytoskeleton and focal adhesion.

### 3.4. Transcriptional Expression Profiles of the Genes Related to Enrichment Pathways

**Figure 7:**
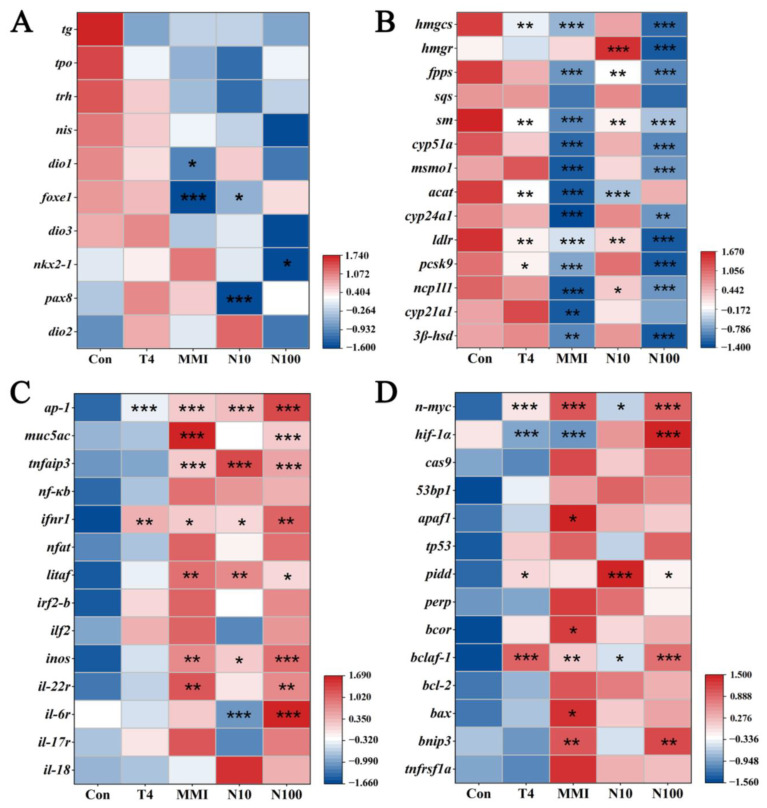
Heatmap of gene expression of the genes involved in (**A**) thyroid hormone metabolism, (**B**) cholesterol metabolism, (**C**) inflammation and immune response, (**D**) and apoptosis. The vertical axis represents the level of gene expression, and the horizontal axis represents different treatment groups. The color of the square represents the level of gene expression, red indicates a relatively high expression and blue indicates a relatively low expression. * *p* < 0.05, ** *p* < 0.01, *** *p* < 0.001.

To further investigate the effect of T4, MMI and nitrate on embryos of *B. gargarizans*, based on functional annotation, genes involved in the thyroid hormone metabolism, cholesterol metabolism, inflammation and immune response, and apoptosis were screened for analysis (Figure 7). Relative to the control, the expression of genes involved in thyroid hormone metabolism and cholesterol metabolism showed an overall decreasing trend after all treatments. Among genes related to thyroid hormone metabolism, *forkhead box protein E1* (*foxe1*) was significantly downregulated in the MMI group and N10 group (ANOVA, *p* < 0.05), *paired box protein Pax-8* (*pax8*) in the N10 group (ANOVA, *p* < 0.001), and *homeobox protein Nkx2-1* (*nkx2-1*) in the N100 group (ANOVA, *p* < 0.001) (Figure 7A). For genes related to cholesterol metabolism, the gene expression levels in the MMI and N100 groups were most significantly downregulated, including *hydroxymethylglutaryl-CoA synthase* (*hmgcs*), *farnesyl pyrophosphate synthase* (*fpps*), *squalene monooxygenase* (*sm*), *lanosterol 14-alpha demethylase* (*cyp51a*), *methylsterol monooxygenase 1* (*msmo1*), *1,25-dihydroxyvitamin D*(*3*) *24-hydroxylase* (*cyp24a1*), *3 beta-hydroxysteroid dehydrogenase type 7* (*3β-hsd7*), *low-density lipoprotein receptor* (*ldlr*), *proprotein convertase subtilisin/kexin type 9* (*pcsk9*) and *Niemann-Pick C1-like protein 1* (*ncp1l1*) (ANOVA, *p* < 0.01) (Figure 7B). Additionally, the levels of *3-hydroxy-3-methylglutaryl-Coenzyme A reductase* (*hmgr*) were significantly decreased in the N100 group (ANOVA, *p* < 0.001) (Figure 7B).

In contrast, after all treatments, there was an overall trend of increased expression of the genes related to inflammation and apoptosis compared to the control group. Among the genes related to inflammation and immune response (Figure 7C), the transcription factor *Activator protein-1* (*ap-1*) and *type I interferon receptor 1* (*ifnr1*) were significantly increased in all the treatment groups (ANOVA, *p* < 0.05). And there was a significant increase in the expression levels of *mucin-5AC* (*muc5ac*), *tumor necrosis factor alpha-induced protein 3* (*tnfaip3*) and *lipopolysaccharide-induced TNF factor* (*litaf*) in the MMI, N10 and N100 groups (ANOVA, *p* < 0.05); *interleukin-22 receptor* (*il-22r*) in the MMI and N100 groups (ANOVA, *p* < 0.01); and *interleukin-6 receptor* (*il-6r*) in the N100 group (ANOVA, *p* < 0.001). For the genes related to apoptosis (Figure 7D), *N-myc proto-oncogene protein* (*n-myc*) and *bcl-2-associated transcription factor 1* (*bclaf-1*) were significantly increased in all the treatment groups (ANOVA, *p* < 0.05). And there was a significant increase in the expression levels of *p53-induced death domain-containing protein* (*pidd*) in the T4, N10 and N100 groups (ANOVA, *p* < 0.05); *BCL2/adenovirus E1B 19 kDa protein-interacting protein 3* (*bnip3*) in the MMI and N100 groups (ANOVA, *p* < 0.01); and *hypoxia-inducible factor 1-alpha* (*hif-1α*) in the N100 group (ANOVA, *p* < 0.001).

## 4. Discussion

Growth parameters, including body length and weight, are among the most sensitive indicators for evaluating developmental toxicity [30,31]. In our study, a significant reduction in total body length and weight was observed after exposure to T4, MMI and N100 on days 7 and 9. Similarly, previous studies showed that T4 and MMI exposure reduced the morphometric parameters of zebrafish embryos [32,33]. Moreover, nitrate has also been reported to inhibit the growth parameters in a variety of aquatic organisms, such as *Gobiocypris rarus* and *Rana sphenocephalus* [18,34]. The inhibition of growth in amphibian embryos often indicates reduced metabolic rates, increased vulnerability to environmental stressors and diminished capacity to evade predators [35,36,37]. Thus, the inhibition of embryonic growth caused by T4, MMI and N100 can negatively impact the viability of the *B. gargarizans* embryos.

In addition, in our study, a significant retardation of embryo development was observed on day 7 of exposure to MMI and N100, whereas the low-concentration N10 group had no significant effect. In contrast, on day 9 of exposure, T4, MMI, N10 and N100 all significantly inhibited embryo development. These findings indicated that the delayed effect of nitrate on embryonic development in *B. gargarizans* is dependent on the dosage and exposure duration. The inhibited growth and delayed development of embryos may be attributed to the disruption of hormone signaling systems and energy metabolism pathways, leading to an insufficient energy supply and compromised growth [38,39]. Nevertheless, further research is necessary to elucidate the underlying metabolic and molecular mechanisms involved.

RNA-seq analysis was conducted to investigate the molecular mechanisms underlying the effects of T4, MMI and nitrate exposures on *B. gargarizans* embryos. As the three exposures have all been reported to interfere with thyroid axis in amphibians [19,29], we initially screened genes related to thyroid hormone metabolism for analysis. In our study, the expression of *foxe1* was significantly decreased in the MMI and N10 groups, *pax8* in the N10 group, and *nkx2-1* in the N100 group. The expression of these three genes plays a considerable role in the proper morphogenesis of the thyroid gland and maintenance of its functionally differentiated state [40]. Specifically, FOXE1 is involved in thyroid precursor cells migration [41], while NKX2-1 and PAX8 are essential for maintaining the survival and structural integrity of thyroid cell precursors [42]. Therefore, we formulated the hypothesis that decreased expression levels of these three genes, respectively, caused by MMI and nitrate, might disrupt morphogenesis of the thyroid gland in the *B. gargarizans* embryos.

In the GO analysis, we found that the top 30 enriched pathways that shared in MMI vs. Con group and N100 vs. Con group were the most compared with other groups, among which we focused on cholesterol metabolic pathways related to energy metabolism. Cholesterol synthesis begins with the conversion of acetyl-CoA to HMG-CoA by the enzyme HMGCS. Mevalonate is then formed through the action of HMGR, which is a rate-limiting step in the pathway. Following a series of enzymatic reactions involving enzymes such as MK, HMGCS, and SQS, squalene is formed. Squalene is ultimately converted into cholesterol through a process of cyclization and subsequent modification, involving enzymes such as CYP51A1 and MSMO1 [43,44]. In our results, almost all these key genes in the cholesterol biosynthesis pathway were significantly downregulated after MMI and N100 exposure, which implies a reduction in cholesterol synthesis. Previous reports have revealed that cholesterol is vital for the integrity, stability and fluidity of cell membranes, it regulates membrane permeability, and facilitating the organization of specialized membrane domains [45,46]. Reduced cholesterol levels in cell membranes can lead to decreased membrane fluidity, increased membrane permeability, hindered formation of lipid rafts and altered membrane protein function [47,48,49]. This can impact various membrane-dependent processes, including impairment of receptor functionality, the alteration of ion channel activity, and the disturbance of membrane trafficking, which can have adverse effects on cellular signaling, nutrient uptake, and overall cellular function [50,51,52]. Therefore, we suggested that the decreased expression levels of genes related to cholesterol synthesis caused by MMI and N100 may have widespread adverse effects on the overall physiology of the *B. gargarizans* embryos through the disruption of membrane structure.

Apart from being an important component of cell membranes, cholesterol is also the precursor of bile acids, vitamin D and several steroid hormones [43]. A reduction in cholesterol synthesis can consequently result in decreased steroid production [53]. Glucocorticoids, a kind of steroid involved in embryonic growth and maturation, play important roles in various physiological processes [54,55]. In our study, genes associated with glucocorticoid synthesis were also significantly downregulated in the MMI and N100 groups, such as *3β-hsd* and *cyp21a1*. Previous studies reported that glucocorticoids modulate glucose metabolism by promoting gluconeogenesis and inhibiting glucose uptake in peripheral tissues, and influence lipid metabolism by promoting lipolysis in adipose tissue [56,57]. In addition, glucocorticoids contribute to the maturation of various organs, they maintain alveolar expansion by stimulating the production of surfactant proteins, and adjust development of liver, kidney, and neural tissues by regulating cellular proliferation and differentiation [54,58,59]. Therefore, we speculated that glucocorticoid deficiency caused by MMI and N100 can lead to impaired energy homeostasis and damage the development of organs, ultimately delaying the embryonic development process of *B. gargarizans*. Consistent with this inference, in our study, MMI and N100 exhibited the earliest and most pronounced inhibitory effects on embryonic development stage.

Glucocorticoids also function as potent anti-inflammatory agents, and a decrease in their expression can result in immunologic dysfunction, increased inflammation, and impaired immune system modulation [60]. MUC5AC is an inflammatory response-induced mucin protein, and its production has been reported to be suppressed by glucocorticoids [61,62]. In our study, the expression level of *muc5ac* was significantly up-regulated in the MMI and N100 groups. Increased MUC5AC production is associated with mucus hypersecretion, which can lead to the formation of thickened mucus layers and impaired clearance of luminal contents [63]. This can interfere with the proper functioning of the digestive and respiratory tracts, leading to impaired nutrient absorption and airway obstruction [64,65]. Besides, excessive mucus production can foster a microenvironment for pathogen growth and colonization, increasing the risk of infection and inflammation in organisms [12,66]. Thus, the elevated expression level of *muc5ac* in *B. gargarizans* embryos after MMI and N100 exposure may disrupt normal physiological processes and increase susceptibility to infections.

Furthermore, we also found that the expression level of *il-22r* was significantly upregulated in the MMI and N100 groups. IL-22R is a cell surface receptor that binds to IL-22, an immune cytokine generated by various immune cells [67]. Previous studies have shown that the increased expression of IL-22R was observed in various types of inflammation involving epithelial tissue and mucosal surfaces, such as inflammatory bowel disease (IBD), atopic dermatitis, and chronic obstructive pulmonary disease (COPD) [68]. In inflammatory diseases, IL-22R-mediated signal transduction maintains tissue homeostasis and mitigates inflammation by facilitating tissue repair, fine-tuning the inflammatory response, and bolstering antimicrobial defense mechanisms [68,69,70]. Therefore, it can be inferred that MMI and N100 exposure treatment may induce the inflammation associated with the mucosal barrier, and the increased expression levels of *il-22r* in *B. gargarizans* embryos indicate potential adaptive responses to inflammation.

Inducible nitric oxide synthase (iNOS) is an enzyme that produces a great deal of nitric oxide (NO) in response to various inflammatory and immune stimuli [71]. Numerous previous studies have shown that NO, as an important inflammatory mediator, is involved in the pathogenesis of various diseases and tissue injuries [72,73]. For example, in mice with hemorrhagic shock, NO produced by iNOS promotes the activation of proinflammatory transcription factors, eliciting an inflammatory response that contributes to organ impairment in the liver and lungs [74]. During sepsis, the increased expression of iNOS leads to excessive release of NO, which causes relaxation of vascular smooth muscle and disruption of calcium homeostasis, leading to ventricular dysfunction [75]. Moreover, excessive NO production suppresses the proliferation and differentiation of T cells, which can lead to immunosuppression and impair the organism’s ability to mount effective immune defenses [76]. In our results, the expression level of *inos* was significantly upregulated in the MMI, N10 and N100 groups. Therefore, we speculated that the upregulation of *inos* expression level after MMI and nitrate exposure would lead to the massive production of NO, which would disrupt the balance of immune response in *B. gargarizans* embryos, and lead to inflammation and tissue damage.

BNIP3, a member of the Bcl-2 protein family, is a pro-apoptotic protein that localizes to the outer mitochondrial membrane [77]. It engages with other proteins to destroy the integrity of the mitochondrial membrane, resulting in the release of Cytochrome c and other pro-apoptotic factors, which in turn activates apoptotic pathways and results in cell death [78]. In the heart, BNIP3-mediated necrosis of cardiac myocytes can contribute to ischemic heart disease, including myocardial infarction [79]. In the lung, activation of BNIP3 can result in necrotic cell death in lung epithelial cells, contributing to the progression of severe lung diseases, such as acute lung injury and acute respiratory distress syndrome (ARDS) [80]. In the liver, an overexpression of BNIP3 induces mitophagy disorder and apoptosis, promoting the development of hepatocellular carcinoma (HCC) [78]. In the current study, the significant upregulation of *bnip3* expression level was observed in MMI and N100 groups; we thus speculate that MMI and N100 may disrupt mitochondrial integrity and function by upregulating *bnip3*, leading to cell death and tissue damage in *B. gargarizans* embryos.

## 5. Conclusions

In the current study, morphological results showed that T4, MMI, N10, and N100 exposures inhibited the growth and development of *B. gargarizans* embryos, among which MMI and N100 demonstrated the earliest and most significant inhibitory effects on embryonic development. Simultaneously, transcriptome analysis of GO and KEGG functional enrichment showed that the top 30 enriched pathways shared by MMI and N100 were the most abundant among the four treatment groups. In addition, after the exposure to MMI and nitrate (especially N100), the expression levels of genes related to thyroid morphogenesis and cholesterol metabolism were significantly downregulated, while the expression levels of genes related to inflammation and apoptosis were significantly upregulated. Based on transcriptomic and morphological analysis, we suggest that the inhibition of MMI and nitrate on the embryonic development of *B. gargarizans* may come from the similar mechanism: they disrupt thyroid morphogenesis, cholesterol and glucocorticoid synthesis, and induce inflammation and apoptosis. These effects may contribute to impaired energy homeostasis and organ development, ultimately delaying the embryonic development process. Collectively, these findings contribute to the understanding of the adverse effects of nitrate on *B. gargarizans* embryos and provide a new insight into the mechanisms of toxicity of amphibian embryos to nitrate exposure on the level of morphological and molecular biology.

## Data Availability

Data are available from authors upon request.

## References

[1] Kazakis N., Matiatos I., Ntona M.-M., Bannenberg M., Kalaitzidou K., Kaprara E., Mitrakas M., Ioannidou A., Vargemezis G., Voudouris K. (2020). Origin, implications and management strategies for nitrate pollution in surface and ground waters of Anthemountas basin based on a δ_15_N-NO_3_^−^ and δ^18^O-NO_3_^−^ isotope approach. Sci. Total Environ..

[2] Yu J., Wang X., Qian S., Liu P., Li X., Li J. (2022). Exposure to nitrate induces alterations in blood parameter responses, liver immunity, and lipid metabolism in juvenile turbot (*Scophthalmus maximus*). Aquat. Toxicol..

[3] Ahmed M., Rauf M., Mukhtar Z., Saeed N.A. (2017). Excessive use of nitrogenous fertilizers: An unawareness causing serious threats to environment and human health. Environ. Sci. Pollut. Res. Int..

[4] Velusamy K., Periyasamy S., Kumar P.S., Vo D.-V.N., Sindhu J., Sneka D., Subhashini B. (2021). Advanced techniques to remove phosphates and nitrates from waters: A review. Environ. Chem. Lett..

[5] Cao Y., Tang C., Song X., Liu C., Zhang Y. (2012). Characteristics of nitrate in major rivers and aquifers of the Sanjiang Plain, China. J. Environ. Monit..

[6] Re V., Sacchi E., Kammoun S., Tringali C., Trabelsi R., Zouari K., Daniele S. (2017). Integrated socio-hydrogeological approach to tackle nitrate contamination in groundwater resources. The case of Grombalia Basin (Tunisia). Sci. Total Environ..

[7] Camargo J.A., Alonso A., Salamanca A. (2005). Nitrate toxicity to aquatic animals: A review with new data for freshwater invertebrates. Chemosphere.

[8] Yu J., Wang Y., Xiao Y., Li X., Zhou L., Wang Y., Du T., Ma X., Li J. (2021). Investigating the effect of nitrate on juvenile turbot (*Scophthalmus maximus*) growth performance, health status, and endocrine function in marine recirculation aquaculture systems. Ecotoxicol. Environ. Saf..

[9] Edwards T.M., Guillette L.J. (2007). Reproductive characteristics of male mosquitofish (*Gambusia holbrooki*) from nitrate-contaminated springs in Florida. Aquat. Toxicol..

[10] Kellock K.A., Moore A.P., Bringolf R.B. (2018). Chronic nitrate exposure alters reproductive physiology in fathead minnows. Environ. Pollut..

[11] Schram E., Roques J.A.C., Abbink W., Yokohama Y., Spanings T., de Vries P., Bierman S., van de Vis H., Flik G. (2014). The impact of elevated water nitrate concentration on physiology, growth and feed intake of African catfish *Clarias gariepinus* (Burchell 1822). Aquac. Res..

[12] Li J., Ye Z. (2020). The potential role and regulatory mechanisms of MUC5AC in chronic obstructive pulmonary disease. Molecules.

[13] Ruthsatz K., Bartels F., Stützer D., Eterovick P.C. (2022). Timing of parental breeding shapes sensitivity to nitrate pollution in the common frog *Rana temporaria*. J. Therm. Biol..

[14] Licht L.E. (2003). Shedding light on ultraviolet radiation and amphibian embryos. BioScience.

[15] Pechenik J.A. (2006). Larval experience and latent effects—Metamorphosis is not a new beginning. Integr. Comp. Biol..

[16] Urbina J., Bredeweg E.M., Blaustein A.R., Garcia T.S. (2021). Direct and latent effects of pathogen exposure across native and invasive amphibian life stages. Front. Vet. Sci..

[17] Hinther A., Edwards T.M., Guillette L.J., Helbing C.C. (2012). Influence of nitrate and nitrite on thyroid hormone responsive and stress-associated gene expression in cultured *Rana catesbeiana* tadpole tail fin tissue. Front. Genet..

[18] Ortiz-Santaliestra M.E., Sparling D.W. (2007). Alteration of larval development and metamorphosis by nitrate and perchlorate in southern leopard frogs (*Rana sphenocephala*). Arch. Environ. Contam. Toxicol..

[19] Wang M., Chai L., Zhao H., Wu M., Wang H. (2015). Effects of nitrate on metamorphosis, thyroid and iodothyronine deiodinases expression in *Bufo gargarizans* larvae. Chemosphere.

[20] Nieuwkoop P.D., Faber J. (1956). Normal Table of Xenopus laevis (Daudin).

[21] Gosner K.L. (1960). A simplified table for staging anuran embryos and larvae with notes on identification. Herpetologica.

[22] Honda J., Ogawa K., Taniguchi K. (1993). Immunohistochemical and morphometric studies on the development of the thyroid, parathyroid and ultimobranchial body in *Xenopus laevis* Daudin. Exp. Anim..

[23] Morvan Dubois G., Sebillot A., Kuiper G.G., Verhoelst C.H., Darras V.M., Visser T.J., Demeneix B.A. (2006). Deiodinase activity is present in *Xenopus laevis* during early embryogenesis. Endocrinology.

[24] Lee C., Fong J.J., Jiang J.-P., Li P.-P., Waldman B., Chong J.R., Lee H., Min M.-S. (2021). Phylogeographic study of the *Bufo gargarizans* species complex, with emphasis on Northeast Asia. Anim. Cells Syst..

[25] Luo Z., Li C., Wang H., Zhao M., Gu Q., Gu Z., Liao C., Wu H. (2014). Mutual mate choice in the Asiatic toad, *Bufo gargarizans*, exerts stabilizing selection on body size. Chin. Sci. Bull..

[26] Othman S.N., Litvinchuk S.N., Maslova I., Dahn H., Messenger K.R., Andersen D., Jowers M.J., Kojima Y., Skorinov D.V., Yasumiba K. (2022). From Gondwana to the Yellow Sea, evolutionary diversifications of true toads *Bufo* sp. in the Eastern Palearctic and a revisit of species boundaries for Asian lineages. Elife.

[27] Niu Z., Liu Y., Wang Y., Liu Y., Chai L., Wang H. (2023). Impairment of bile acid metabolism and altered composition by lead and copper in *Bufo gargarizans* tadpoles. Sci. Total Environ..

[28] Crane H.M., Pickford D.B., Hutchinson T.H., Brown J.A. (2006). The effects of methimazole on development of the fathead minnow, *Pimephales promelas*, from embryo to adult. Toxicol. Sci..

[29] Fabrezi M., Cruz J.C. (2021). Phenotypic variation through ontogeny: Thyroid axis disruption during larval development in the frog *Pleurodema borellii*. Front. Ecol. Evol..

[30] Brunelli E., Bernabò I., Berg C., Lundstedt-Enkel K., Bonacci A., Tripepi S. (2009). Environmentally relevant concentrations of endosulfan impair development, metamorphosis and behaviour in *Bufo bufo* tadpoles. Aquat. Toxicol..

[31] Eriyamremu G.E., Osagie V.E., Omoregie S.E., Omofoma C.O. (2008). Alterations in glutathione reductase, superoxide dismutase, and lipid peroxidation of tadpoles (*Xenopus laevis*) exposed to Bonny Light crude oil and its fractions. Ecotoxicol. Environ. Saf..

[32] Godfrey A., Hooser B., Abdelmoneim A., Horzmann K.A., Freemanc J.L., Sepúlveda M.S. (2017). Thyroid disrupting effects of halogenated and next generation chemicals on the swim bladder development of zebrafish. Aquat. Toxicol..

[33] Santos T.P., da Silva Bastos P.E., da Silva J.F., de Medeiros Vieira S.M., da Silva M.C.G., de Andrade A.L.C., Padilha R.M.O., dos Santos Magnabosco A.R., Cadena M.R.S., Cadena P.G. (2023). Single and joint toxic effects of thyroid hormone, levothyroxine, and amiodarone on embryo-larval stages of zebrafish (*Danio rerio*). Ecotoxicology.

[34] Luo S., Wu B., Xiong X., Wang J. (2016). Short-term toxicity of ammonia, nitrite, and nitrate to early life stages of the rare minnow (*Gobiocypris rarus*). Environ. Toxicol. Chem..

[35] Allran J., Karasov W. (2000). Effects of atrazine and nitrate on northern leopard frog (*Rana pipiens*) larvae exposed in the laboratory from posthatch through metamorphosis. Environ. Toxicol. Chem..

[36] Berven K.A. (1990). Factors affecting population fluctuations in larval and adult stages of the wood frog (*Rana sylvatica*). Ecology.

[37] Smith D.C. (1987). Adult recruitment in chorus frogs: Effects of size and date at metamorphosis. Ecology.

[38] Liu Y., Wang H., Chai L., Li X., Wu M., Wang H. (2022). Effects of perchlorate and exogenous T4 exposures on development, metamorphosis and endochondral ossification in *Bufo gargarizans* larvae. Aquat. Toxicol..

[39] Rowe C.L., Kinney O.M., Nagle R.D., Congdon J.D. (1998). Elevated maintenance costs in an anuran (*Rana catesbeiana*) exposed to a mixture of trace elements during the embryonic and early larval periods. Physiol. Zool..

[40] Fernández L.P., López-Márquez A., Santisteban P. (2015). Thyroid transcription factors in development, differentiation and disease. Nat. Rev. Endocrinol..

[41] Gandarilla-Esparza D.D., Calleros-Rincón E.Y., Macias H.M., González-Delgado M.F., Vargas G.G., Sustaita J.D., González-Zamora A., Ríos-Sánchez E., Pérez-Morales R. (2021). FOXE1 polymorphisms and chronic exposure to nitrates in drinking water cause metabolic dysfunction, thyroid abnormalities, and genotoxic damage in women. Genet. Mol. Biol..

[42] De Felice M., Di Lauro R. (2004). Thyroid development and its disorders: Genetics and molecular mechanisms. Endocr. Rev..

[43] Cerqueira N.M.F.S.A., Oliveira E.F., Gesto D.S., Santos-Martins D., Moreira C., Moorthy H.N., Ramos M.J., Fernandes P.A. (2016). Cholesterol Biosynthesis: A Mechanistic Overview. Biochemistry.

[44] Kovacs W.J., Olivier L.M., Krisans S.K. (2002). Central role of peroxisomes in isoprenoid biosynthesis. Prog. Lipid Res..

[45] Olżyńska A., Kulig W., Mikkolainen H., Czerniak T., Jurkiewicz P., Cwiklik L., Rog T., Hof M., Jungwirth P., Vattulainen I. (2020). Tail-oxidized cholesterol enhances membrane permeability for small solutes. Langmuir.

[46] Simons K., Ikonen E. (2000). How cells handle cholesterol. Science.

[47] Fernández-Pérez E.J., Sepúlveda F.J., Peters C., Bascuñán D., Riffo-Lepe N.O., González-Sanmiguel J., Sánchez S.A., Peoples R.W., Vicente B., Aguayo L.G. (2018). Effect of cholesterol on membrane fluidity and association of Aβ oligomers and subsequent neuronal damage: A double-edged sword. Front. Aging Neurosci..

[48] Lee A.G. (2004). How lipids affect the activities of integral membrane proteins. BBA—Biomembr..

[49] Paukner K., Králová Lesná I., Poledne R. (2022). Cholesterol in the cell membrane-An emerging player in atherogenesis. Int. J. Mol. Sci..

[50] Ammendolia D.A., Bement W.M., Brumell J.H. (2021). Plasma membrane integrity: Implications for health and disease. BMC Biol..

[51] Papalazarou V., Maddocks O.D.K. (2021). Supply and demand: Cellular nutrient uptake and exchange in cancer. Mol. Cell.

[52] Sunshine H., Iruela-Arispe M.L. (2017). Membrane lipids and cell signaling. Curr. Opin. Lipidol..

[53] Rone M.B., Fan J., Papadopoulos V. (2009). Cholesterol transport in steroid biosynthesis: Role of protein–protein interactions and implications in disease states. Biochim. Biophys. Acta (BBA)—Mol. Cell Biol. Lipids.

[54] Bablok M., Gellisch M., Scharf M., Brand-Saberi B., Morosan-Puopolo G. (2023). Spatiotemporal expression pattern of the chicken glucocorticoid receptor during early embryonic development. Ann. Anat..

[55] Fowden A.L., Forhead A.J. (2015). Glucocorticoids as regulatory signals during intrauterine development. Exp. Physiol..

[56] Kuo T., McQueen A., Chen T.-C., Wang J.-C., Wang J.-C., Harris C. (2015). Regulation of glucose homeostasis by glucocorticoids. Glucocorticoid Signaling: From Molecules to Mice to Man.

[57] Vegiopoulos A., Herzig S. (2007). Glucocorticoids, metabolism and metabolic diseases. Mol. Cell. Endocrinol..

[58] Seckl J.R., Holmes M.C. (2007). Mechanisms of Disease: Glucocorticoids, their placental metabolism and fetal ‘programming’ of adult pathophysiology. Nat. Clin. Pract. Endocrinol. Metab..

[59] Stubbe J., Madsen K., Nielsen F.T., Skøtt O., Jensen B.L. (2006). Glucocorticoid impairs growth of kidney outer medulla and accelerates loop of Henle differentiation and urinary concentrating capacity in rat kidney development. Am. J. Physiol.-Ren. Physiol..

[60] Sarapultsev A., Sarapultsev P., Dremencov E., Komelkova M., Tseilikman O., Tseilikman V. (2020). Low glucocorticoids in stress-related disorders: The role of inflammation. Stress.

[61] NandyMazumdar M., Bagchi D., Das A., Downs B.W. (2023). Chapter 2—Airway mucus, infection, and therapeutic strategies. Viral, Parasitic, Bacterial, and Fungal Infections.

[62] Takami S., Mizuno T., Oyanagi T., Tadaki H., Suzuki T., Muramatsu K., Takizawa T., Arakawa H. (2012). Glucocorticoids inhibit MUC5AC production induced by transforming growth factor-α in human respiratory cells. Allergol. Int..

[63] Evans C.M., Kim K., Tuvim M.J., Dickey B.F. (2009). Mucus hypersecretion in asthma: Causes and effects. Curr. Opin. Pulm. Med..

[64] Cornick S., Tawiah A., Chadee K. (2015). Roles and regulation of the mucus barrier in the gut. Tissue Barriers.

[65] Fahy J.V., Dickey B.F. (2010). Airway mucus function and dysfunction. N. Engl. J. Med..

[66] Rogers D. (2009). Airway mucus hypersecretion in asthma and COPD. Asthma and COPD: Basic Mechanisms and Clinical Management.

[67] Liang S.C., Nickerson-Nutter C., Pittman D.D., Carrier Y., Goodwin D.G., Shields K.M., Lambert A.J., Schelling S.H., Medley Q.G., Ma H.L. (2010). IL-22 induces an acute-phase response. J. Immunol..

[68] Sabat R., Ouyang W., Wolk K. (2014). Therapeutic opportunities of the IL-22–IL-22R1 system. Nat. Rev. Drug Discov..

[69] Aujla S.J., Chan Y.R., Zheng M., Fei M., Askew D.J., Pociask D.A., Reinhart T.A., McAllister F., Edeal J., Gaus K. (2008). IL-22 mediates mucosal host defense against Gram-negative bacterial pneumonia. Nat. Med..

[70] Yan J., Yu J., Liu K., Liu Y., Mao C., Gao W. (2021). The pathogenic roles of IL-22 in colitis: Its transcription regulation by musculin in T helper subsets and innate lymphoid cells. Front. Immunol..

[71] Cinelli M.A., Do H.T., Miley G.P., Silverman R.B. (2020). Inducible nitric oxide synthase: Regulation, structure, and inhibition. Med. Res. Rev..

[72] Bogdan C. (2001). Nitric oxide and the immune response. Nat. Immunol..

[73] Sharma J.N., Al-Omran A., Parvathy S.S. (2007). Role of nitric oxide in inflammatory diseases. Inflammopharmacology.

[74] Hierholzer C., Harbrecht B., Menezes J.M., Kane J., MacMicking J., Nathan C.F., Peitzman A.B., Billiar T.R., Tweardy D.J. (1998). Essential role of induced nitric oxide in the initiation of the inflammatory response after hemorrhagic shock. J. Exp. Med..

[75] Greer J. (2015). Pathophysiology of cardiovascular dysfunction in sepsis. BJA Educ..

[76] Sato K., Ozaki K., Oh I., Meguro A., Hatanaka K., Nagai T., Muroi K., Ozawa K. (2007). Nitric oxide plays a critical role in suppression of T-cell proliferation by mesenchymal stem cells. Blood.

[77] Zhang J., Ney P.A. (2009). Role of BNIP3 and NIX in cell death, autophagy, and mitophagy. Cell Death Differ..

[78] Gao A., Jiang J., Xie F., Chen L. (2020). Bnip3 in mitophagy: Novel insights and potential therapeutic target for diseases of secondary mitochondrial dysfunction. Clin. Chim. Acta.

[79] Regula K.M., Ens K., Kirshenbaum L.A. (2002). Inducible expression of BNIP3 provokes mitochondrial defects and hypoxia-mediated cell death of ventricular myocytes. Circ. Res..

[80] Kim J.-Y., Kim Y.-J., Lee S., Park J.-H. (2011). BNip3 is a mediator of TNF-induced necrotic cell death. Apoptosis.

